# Warburg Effect’s Manifestation in Aggressive Pheochromocytomas and Paragangliomas: Insights from a Mouse Cell Model Applied to Human Tumor Tissue

**DOI:** 10.1371/journal.pone.0040949

**Published:** 2012-07-31

**Authors:** Stephanie M. J. Fliedner, Nina Kaludercic, Xiao-Sheng Jiang, Hana Hansikova, Zuzana Hajkova, Jana Sladkova, Andrea Limpuangthip, Peter S. Backlund, Robert Wesley, Lucia Martiniova, Ivana Jochmanova, Nikoletta K. Lendvai, Jan Breza, Alfred L. Yergey, Nazareno Paolocci, Arthur S. Tischler, Jiri Zeman, Forbes D. Porter, Hendrik Lehnert, Karel Pacak

**Affiliations:** 1 Program in Reproductive and Adult Endocrinology, Eunice Kennedy Shriver National Institute of Child Health and Human Development, National Institutes of Health, Bethesda, Maryland, United States of America; 2 Division of Cardiology, Department of Medicine, Johns Hopkins Medical Institutions, Baltimore, Maryland, United States of America; 3 Section on Molecular Dysmorphology, Eunice Kennedy Shriver National Institute of Child Health and Human Development, National Institutes of Health, Bethesda, Maryland, United States of America; 4 Department of Pediatrics and Adolescent Medicine, First Faculty of Medicine, Charles University and General University Hospital in Prague, Prague, Czech Republic; 5 Section on Mass Spectrometry and Metabolism, Eunice Kennedy Shriver National Institute of Child Health and Human Development, National Institutes of Health, Bethesda, Maryland, United States of America; 6 Warren G. Magnuson Clinical Center, National Institutes of Health, Bethesda, Maryland, United States of America; 7 1st Department of Internal Medicine Medical Faculty, P.J.Šafárik University, Košice, Slovakia; 8 Department of Urology, School of Medicine, Comenius University, Bratislava, Slovakia; 9 Department of Clinical Medicine, Section of Pathology, University of Perugia, Perugia, Italy; 10 Department of Pathology, Tufts Medical Center, Boston, Massachusetts, United States of America; 11 1^st^ Department of Medicine, University Hospitals of Schleswig-Holstein, Lübeck, Germany; Dana-Farber Cancer Institute, United States of America

## Abstract

A glycolytic profile unifies a group of pheochromocytomas and paragangliomas (PHEOs/PGLs) with distinct underlying gene defects, including von Hippel-Lindau (VHL) and succinate dehydrogenase B (SDHB) mutations. Nevertheless, their tumor aggressiveness is distinct: PHEOs/PGLs metastasize rarely in VHL-, but frequently in SDHB-patients. To date, the molecular mechanisms causing the more aggressive phenotype in SDHB-PHEOs/PGLs remain largely unknown. Recently, however, an excellent model to study aggressive PHEOs (mouse tumor tissue (MTT) cells) has been developed from mouse PHEO cells (MPC). We employed this model for a proteomics based approach to identify changes characteristic for tumor aggressiveness, which we then explored in a homogeneous set of human SDHB- and VHL-PHEOs/PGLs. The increase of glucose transporter 1 in VHL, and of hexokinase 2 in VHL and SDHB, confirmed their glycolytic profile. In agreement with the cell model and in support of decoupling of glycolysis, the Krebs cycle and oxidative phosphorylation (OXPHOS), SDHB tumors showed increased lactate dehydrogenase levels. In SDHB-PGLs OXPHOS complex activity was increased at complex III and, as expected, decreased at complex II. Moreover, protein and mRNA expression of all tested OXPHOS-related genes were higher in SDHB- than in VHL-derived tumors. Although there was no direct evidence for increased reactive oxygen species production, elevated superoxide dismutase 2 expression may reflect elevated oxidative stress in SDHB-derived PHEOs/PGLs. For the first time, we show that despite dysfunction in complex II and evidence for a glycolytic phenotype, the Warburg effect does not seem to fully apply to SDHB-PHEOs/PGLs with respect to decreased OXPHOS. In addition, we present evidence for increased LDHA and SOD2 expression in SDHB-PHEOs/PGLs, proteins that have been proposed as promising therapeutic targets in other cancers. This study provides new insight into pathogenic mechanisms in aggressive human PHEOs/PGLs, which may lead to identifying new diagnostic and prognostic markers in the near future.

## Introduction

After going unnoticed for decades, Warburg’s hypothesis of a glycolytic phenotype in tumors is now increasingly recognized. Particularly in highly aggressive tumors a shift from efficient ATP synthesis via oxidative phosphorylation (OXPHOS) to increased glycolysis has been observed [1], [2], [3]. These changes in energy metabolism have been linked to increased angiogenesis, impaired apoptosis, and generation of an acidic tumor environment [4]. Increased glycolysis has been shown to result from suppression of OXPHOS due to a hypoxic state in tumor areas with poor blood and oxygen supply, from mutation of key regulatory genes, or from increased reactive oxygen species (ROS) levels. The latter two lead to a pseudo-hypoxic state under normoxic conditions [5], [6], [7], [8]. Hypoxia has been recognized as a predictive marker for metastatic disease, therapy resistance, and poor outcome in several types of cancer [9], [10].

Recently, mutations of a group of key regulatory genes causing pseudo-hypoxia and a glycolytic phenotype – i.e. the nuclear encoded mitochondrial succinate dehydrogenase (SDH) subunits A, B, C, and D – were recognized as tumorigenic. Mutations of the SDHx genes can cause pheochromocytomas (PHEOs) and paragangliomas (PGLs) [11], [12], [13], [14]. Thus, SDHx-derived PHEOs/PGLs represent a unique type of cancer for studying the Warburg effect and its role in aggressive tumors because of the impaired OXPHOS and/or Krebs cycle in these tumors.

In SDHx-related PHEOs/PGLs, the dual roles of the SDH complex seem to decrease OXPHOS related ATP synthesis. First, its function as complex II in the electron transfer chain of the OXPHOS is impaired, resulting in decreased activity in SDHB and D [15], [16], [17], [18], and possibly in A and C, mutations. Second, the SDH complex catalyzes conversion of succinate into fumarate in the Krebs cycle. Accumulation of succinate and/or possibly ROS, due to enzyme malfunction, leads to inhibition of hypoxia-inducible factor (HIF) prolyl hydroxylases [19], [20]. Under normoxia HIF prolyl hydroxylases flag HIFαs for recognition by an E3 ligase complex containing the von Hippel-Lindau protein (pVHL). This complex then facilitates appropriate degradation of HIFαs under normoxia. When SDH is malfunctioning, HIF prolyl hydroxylases cannot adequately tag HIFα subunits for this proteasomal degradation. Stabilized HIFα subunits then assemble with the constitutively expressed β subunits and together they act as transcription factors. HIF-responsive genes are activated, with strengthening of glycolysis, while OXPHOS activity, particularly complex II expression, is decreased [16], [21].

Another type of PHEOs/PGLs, namely those derived due to VHL mutations, share the pseudo-hypoxic/glycolytic phenotype [21]. The study of VHL-derived tumors initially led to the discovery of pseudo-hypoxia and its supporting role in tumor growth [22]. Dysfunction or absence of pVHL leads to inhibition of HIFα degradation and consequently pseudo-hypoxia (for review: [23]).

Despite their similarities in presenting with pseudo-hypoxic/glycolytic phenotypes, tumor aggressiveness in SDHx- and VHL-derived PHEOs/PGLs is distinct. Development of metastases in patients with VHL-, SDHA-, C-, and D-derived PHEOs/PGLs is rare, while patients with SDHB-related PHEOs/PGLs frequently present with metastatic disease [24], [25], [26], [27], [28]. To date, aggressive tumor behavior of PHEOs/PGLs has not been conclusively studied with respect to energy metabolism. This is partially due to several limitations, including: limited availability of SDHB-related tumors, unsuccessful establishment to date of an SDHB cell line, difficulties in maintaining primary cells from human PHEOs/PGLs in culture, and the absence of an SDHB animal model.

Recently, mouse PHEO cells (MPC) were established as an excellent tool for the study of the molecular biology of PHEO *in vitro* and *in vivo* [29], [30]. Tumors identical with PHEOs/PGLs develop after i.v. and s.c. injection of these cells into nude mice. Martiniova et al. recently developed the more aggressive mouse tumor tissue (MTT) cells [31] by bulk culture of a liver tumor that developed after tail vein injection of MPC into a nude mouse. Magnetic resonance imaging and microarray gene expression profiling comparing MPC to MTT cells revealed changes reflective of more aggressive behavior of the latter. Thus, comparison of the molecular biology of MTT cells to MPC may provide valuable insight into causes for aggressive behavior in PHEOs/PGLs. By focusing on the cell model, typical inter-patient variability in gene expression can be avoided, possibly allowing for an unbiased view onto changed molecular pathways. Therefore, we decided to run a comparative protein expression study of MPC and MTT cells as a first step to detect proteins that seem to be of importance for an aggressive PHEO/PGL phenotype. Analysis of the differentially expressed proteins focused on changes in energy metabolism related pathways. Expression changes detected in the MPC and MTT cells were then evaluated in human VHL- and SDHB-derived PHEOs/PGLs. Changes related to energy metabolism were further evaluated by measurement of OXPHOS complex activity, ROS production, and expression analysis of additional glycolysis and OXPHOS related genes. The results presented here may lead to better characterization and understanding of the pathogenesis of aggressive PHEOs/PGLs.

## Results

### Two-dimensional-gels of MPC and MTT Cells and Validation

Image analysis of the silver stained two-dimensional-(2D)-gels revealed different intensities (fold change>1.5, p<0.05) for 127 spots. Spots of interest were narrowed down to 46, based on a scoring system considering spot intensity, ratio between groups and consistency within replicates. Protein identification was successful for 38 of the 46 spots, and revealed 53 different proteins. A list of proteins identified for each of the 38 spots, identification specifics, and fold changes of the spots are given in Table S1. The peptide sequences used to identify the proteins are listed in the supplemental material along with their database search scores (Table S2). The mass spectrometry data associated with this manuscript may be downloaded from ProteomeCommons.org Tranche using the following hash:

VyhyibjCNhEsaTH3O33n79p22yl69bq42ElQdBzetPGSbDFObRov3E2OVnWJo6yX8PpVUzozggXkyjd/ANpbCpcEYrwAAAAAAABZIQ =  = .

The hash may be used to prove exactly what files were published as part of this manuscript’s data set, and the hash may also be used to check that the data has not changed since publication.

**Figure 1:**
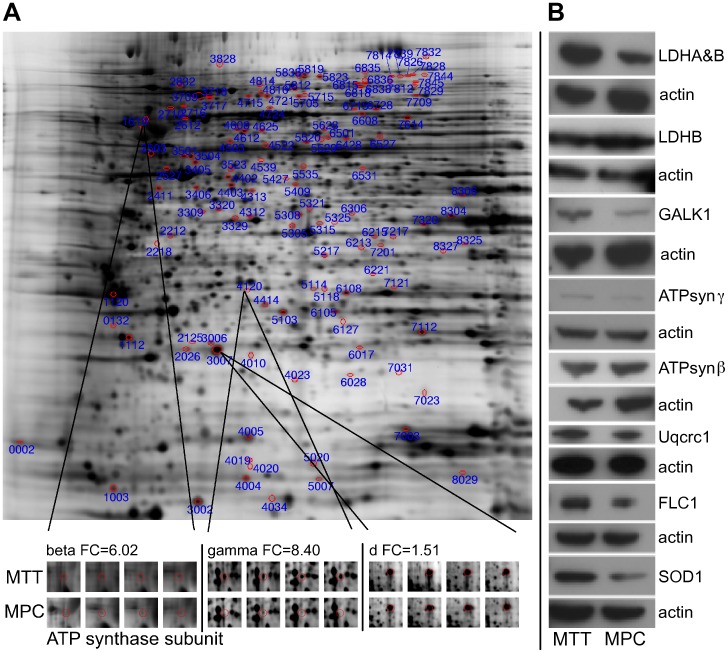
Protein expression in MPC compared to MTT. **A** Representative silver staining of 2D-gel. Spots of interest are circled in red. Magnification of spots containing ATP synthase subunits are shown below for all four MPC and MTT gels. **B** Western blot for validation of differential expression of selected proteins of interest.

**Figure 2:**
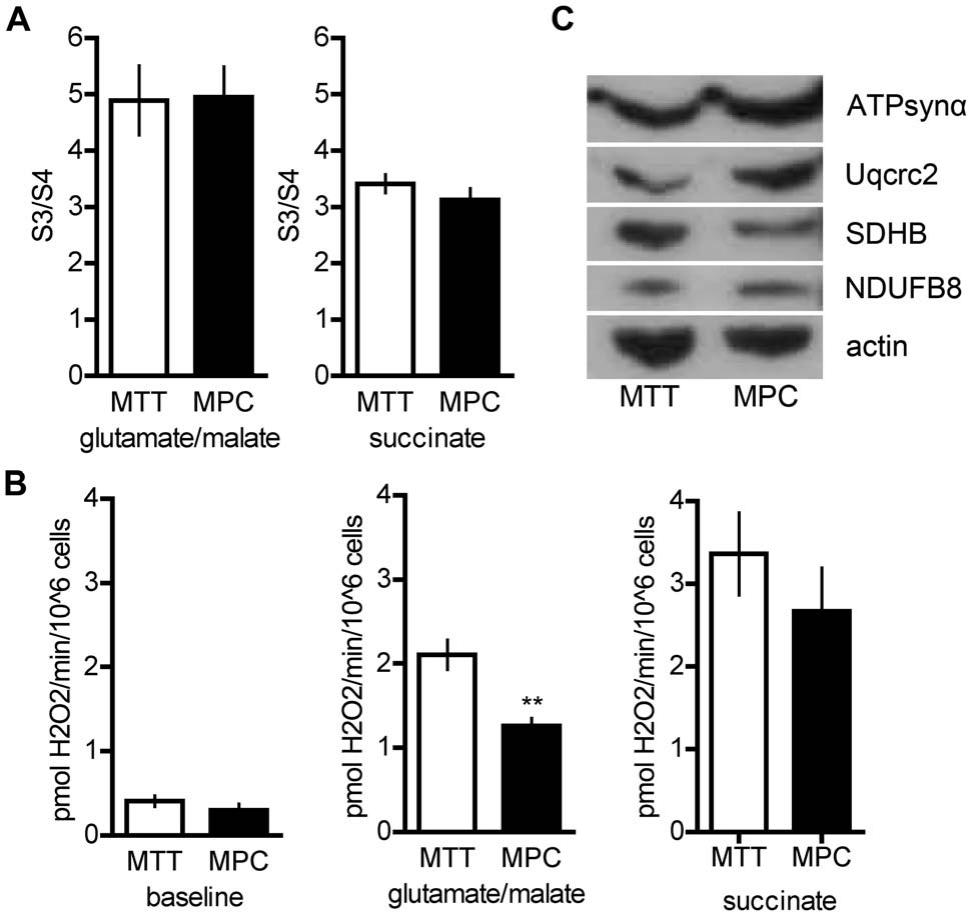
Oxidative phosphorylation complex activity, reactive oxygen species production, and expression of selected oxidative phosphorylation complex subunits in MPC and MTT. A Ratio of ADP-stimulated (S3) and baseline oxygen consumption (S4) in MPC and MTT. **B** Baseline hydrogen peroxide levels (left) and those observed after addition of substrate (glutamate/malate, complex I (center); succinate, complex II (right). ** indicates p<0.01. **C** protein expression of selected subunits of OXPHOS complexes (complex I: NADH dehydrogenase 1 beta subcomplex subunit 8 (NDUFB8); complex II: succinate dehydrogenase B (SDHB); complex III: ubiquinol-cytochrome-c reductase complex core protein 2 (Uqcrc2); complex V: ATP synthase α (ATPsynα)).

Ingenuity Pathway Analysis of the identified proteins and the fold changes of their spot intensities revealed the OXPHOS pathway as the canonical pathway with the most significant changes. Four identified spots contained subunits of OXPHOS complexes: the ubiquinol-cytochrome-c reductase complex core protein 1 (Uqcrc1, OXPHOS complex III, MPC>MTT, Q9CZ13, spot 3405), ATP synthase β, γ, and d subunits (OXPHOS complex V, MTT>MPC, P56480, spot 1619; Q91VR2, spot 4120; Q9DCX2, spot 3007) (Fig. 1A). Except for the spot representing ATP synthase d, each of these spots contained additional proteins that potentially influenced the change of intensity between the groups (Table S1). An increased protein level of ATP synthase γ in MTT cells was verified by western blot, while ATP synthase α and β as well as Uqcrc1 did not show a difference in protein level (Fig. 1B). However, western blot for Uqcrc2 showed decreased expression in MTT cells (Fig. 2C).

The glycolysis related protein lactate dehydrogenase B (LDHB, P16125, spot 4312) was identified from a spot with decreased intensity in MTT cells (fold change = 1.9). Western blot for LDHB verified lower expression in the more aggressive MTT cells. However, western blot for LDH using an antibody that recognized LDHA (P06151) as well as LDHB showed higher signal intensity in MTT cells (Fig. 1B). Thus, overall LDH expression was elevated in MTT cells, with an increased ratio of LDHA to LDHB. Galactokinase 1 (GALK1, Q9R0N0, spot 2411), which is involved in generating glucose from galactose, was identified as differentially expressed (fold change 1.5). Western blot for GALK1 verified higher expression in MTT cells (Fig. 1B). Three other proteins identified from the same spot were not followed up on.

In addition to changes in expression of energy metabolism related proteins, changes in expression of proteins related to ROS production were apparent. Ferritin light chain 1 (FLC1, P29391) as well as copper/zinc superoxide dismutase (SOD1, P08228) were identified from the spot with the highest fold change between the two cell types (spot 4020). FLC1 and SOD1 were also identified from additional spots, with higher intensity in MTT cells than MPC (SOD1: 5020, FLC1: 4010, 4019 (for spot 4019 protein identification with 99% probability was determined using peptide identification at 80%, instead of 95% probability)). With the help of Protein Processor, FLC1 truncation could be identified for the lower molecular weight spots (4019 and 4020), based on absence of peptide fragments that were present in the full size FLC1 (spot 4010). Western blot for both SOD1 and FLC1 verified higher expression in the MTT cells (Fig. 1B).

### Mitochondrial Oxygen Consumption and ROS Production in MPC and MTT Cells

To evaluate if OXPHOS is impaired in MTT cells, mitochondrial oxygen consumption was assessed in digitonin-permeabilized cells. No significant differences were observed between the cell lines in either basal state oxygen consumption or ADP-induced oxygen consumption, using either glutamate/malate (complex I) or succinate (complex II) as substrate (Fig. 2A). However, H_2_O_2_ production with glutamate/malate as substrate was increased, suggesting a non-optimal OXPHOS activity at the level of complex I in MTT cells (Fig. 2B). Interestingly, protein expression of NADH dehydrogenase 1 beta subcomplex subunit 8 (NDUFB8, O95169, complex I) appeared decreased while expression of SDHB (P21912, complex II) appeared increased in MTT cells relative to actin (Fig. 2C). As mentioned above, Uqcrc2 (P22695, complex III) protein level was decreased in MTT cells, while ATP synthase α (ATPsynα, P25705, complex V) appeared unchanged (Fig. 2C).

### Differential Expression in Human PHEOs/PGLs

In the more aggressive MTT cells, we found an increase in overall LDH due to an increased expression of LDHA, while LDHB expression was decreased compared to MPC. We evaluated the expression of LDHA (P00338) and B (P07195) in tumor tissue by qRT-PCR with specific primers for LDHA and B as well as by 2 western blots: one with an antibody recognizing LDHA and B, and one specific for LDHB. The LDHA and B as well as LDHB band intensities were normalized to actin expression on the respective membranes using image J software.

**Figure 3:**
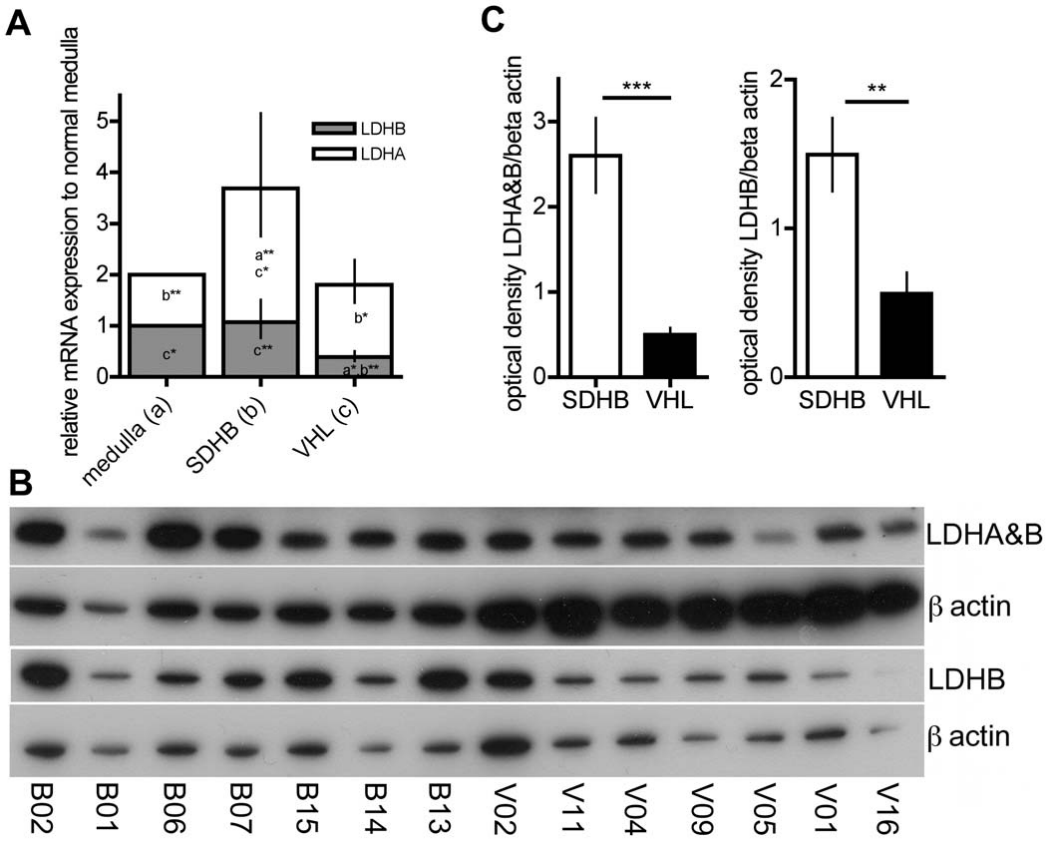
Lactate dehydrogenase expression. **A** Expression of LDHA and LDHB mRNA in SDHB- and VHL-derived PHEO/PGL tissues relative to normal adrenal medulla. A letter and asterisk(s) within each column indicate significant expression differences between the groups were appropriate (a: significantly different from normal medulla, b: significantly different from SDHB, c: significantly different from VHL, *: p<0.05, **: p<0.01). **B** Western blot for LDHA and B as well as LDHB for SDHB-derived tumors (left) and VHL-derived tumors (right). **C** Optical density of bands as assessed with image J software for LDHA and B as well as LDHB from western blots shown in **B**. Significant differences are indicated by *** for p<0.001 and ** for p<0.01.

The mRNA ratio of LDHA to B did not differ between SDHB- and VHL-derived PHEOs/PGLs. However, the ratio was significantly higher in both VHL- and SDHB-derived PHEO/PGL tissues compared to normal adrenal medulla (p = 0.031 and p = 0.012, respectively) (Fig. 3A). LDHA mRNA levels in SDHB were significantly higher than in either normal adrenal medulla or VHL (p = 0.002 and p = 0.036, respectively), while there was no difference between VHL and normal adrenal medulla (Fig. 3A). LDHB mRNA levels were significantly decreased comparing VHL to normal adrenal medulla and SDHB (p = 0.023 and p = 0.001, respectively) (Fig. 3A). In agreement with the mRNA, the ratio of LDHA and B to LDHB did not significantly differ between VHL- and SDHB-derived PHEOs/PGLs on the protein level. However, the relative optical density of LDHA and B as well as LDHB alone was significantly higher in SDHB- than VHL-derived tissue (p<0.001 and p<0.01, respectively) (Fig. 3B, C). Thus, while both tumor types show an increased ratio of LDHA to B, SDHB-derived tumors show a significantly increased overall expression of LDH.

**Figure 4:**
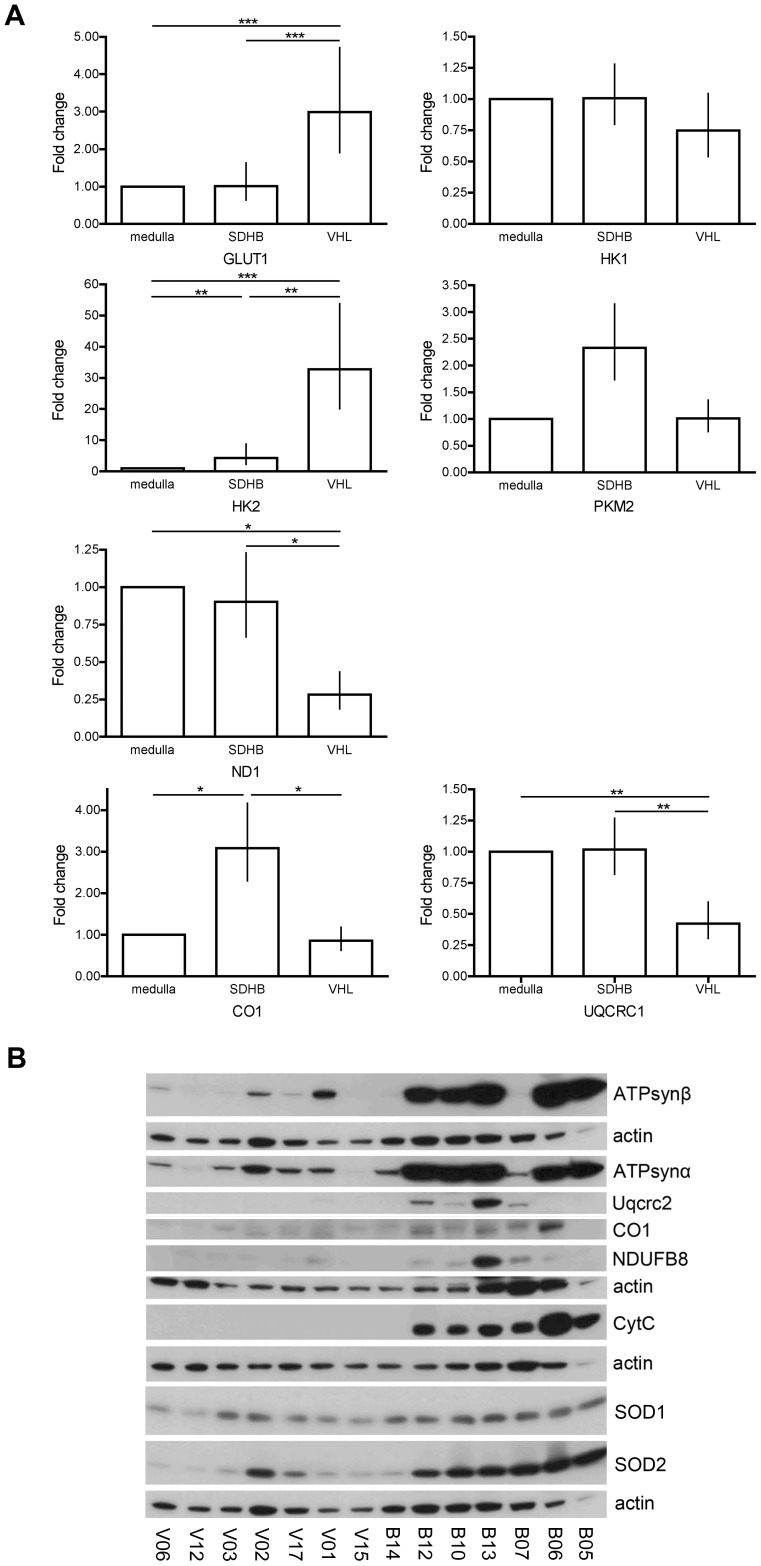
Expression of selected glycolysis, oxidative phosphorylation, and oxidative stress related genes. A mRNA expression of glucose transporter 1 (GLUT1), hexokinase-1 (HK1), hexokinase-2 (HK2), pyruvate kinase isozyme M2 (PKM2), NADH-ubiquinone oxidoreductase chain 1 (ND1), ubiquinol-cytochrome-c reductase complex core protein (UQCRC1), cytochrome c oxidase subunit 1 (CO1) in SDHB- and VHL-derived PHEOs/PGLs relative to normal adrenal medulla. Significant differences are indicated where appropriate by * for p<0.05, ** for p<0.01, and *** for p<0.001. **B** Western blot of selected proteins in SDHB- and VHL-derived PHEOs/PGLs (complex I: NADH dehydrogenase 1 beta subcomplex subunit 8 (NDUFB8); complex III: ubiquinol-cytochrome-c reductase complex core protein 2 (Uqcrc2); complex IV: CO1; complex V: ATP synthase (ATPsyn) α and β, cytochrome C (CytC), superoxide dismutase (SOD) 1, and 2).

In addition, the mRNA levels of the glucose metabolism related glucose transporter type 1 (GLUT1, P11166), hexokinase-1 (HK1, P19367), hexokinase-2 (HK2, P52789), and pyruvate kinase isoenzyme M2 (PKM2, P14618) were determined (Fig. 4A). GLUT1 mRNA expression was elevated in VHL- compared to SDHB-derived and normal adrenal medulla tissue (p<0.001 for both). HK1 mRNA levels showed no differences among the groups, while HK2 expression was elevated 4.3 fold in SDHB- and 32.8 fold in VHL-derived PHEOs/PGLs in comparison to normal adrenal medulla (SDHB vs. medulla: p = 0.006, VHL vs. medulla: p<0.001, VHL vs. SDHB: p = 0.007). PKM2 mRNA expression appeared possibly elevated in SDHB- compared to VHL-derived tumors and normal adrenal medulla. However, the overall ANOVA was not significant (p = 0.124) (Fig. 4A). The Student-Newman-Keuls post-hoc test showed a borderline significant difference between SDHB and VHL (p = 0.066).

Furthermore, the mRNA expression of selected OXPHOS complex subunits was evaluated and revealed significantly decreased levels of NADH-ubiquinone oxidoreductase chain 1 (ND1, P03886, complex I) and Uqcrc1 (P31930, complex III) in VHL tissue, when compared to normal adrenal medulla (p<0.05 and p<0.01, respectively) and to SDHB-derived tissues (p<0.05 and p = 0.01). No difference in mRNA expression between normal adrenal medulla and SDHB-derived PGLs was observed for these two genes. For cytochrome c oxidase subunit 1 (CO1, P00395, complex IV), mRNA expression was significantly increased in SDHB-derived PGLs compared to both normal medulla (p<0.05) and VHL-derived PHEOs (p<0.05). No difference in expression was observed between VHL-derived PHEOs and normal adrenal medulla. While all three genes appear to be expressed more highly in SDHB- than VHL-derived PHEOs/PGLs, ND1 and Uqcrc1 expression appears to be decreased in VHL-derived tumor tissues and close to normal in SDHB-derived PGLs, while CO1 is elevated in SDHB-derived PGLs compared to normal medulla and VHL-derived PHEOs. In support of an elevated expression of selected OXPHOS subunits, protein levels appeared elevated in at least 4 out of 7 SDHB-derived tumors compared to VHL-derived PHEOs for NDUFB8 (O95169, complex I), Uqcrc2 (P22695, complex III), CO1 (complex IV), ATP synthase α (P25705) and β (P06576) (Fig. 4B). In addition, western blot showed a significant increase in expression of the electron carrier cytochrome C (P99999) in SDHB-derived PHEOs/PGLs compared to the VHL samples (Fig. 4B).

### Mitochondrial Electron Transfer Chain Complex Activity in Human PHEOs/PGLs

**Figure 5:**
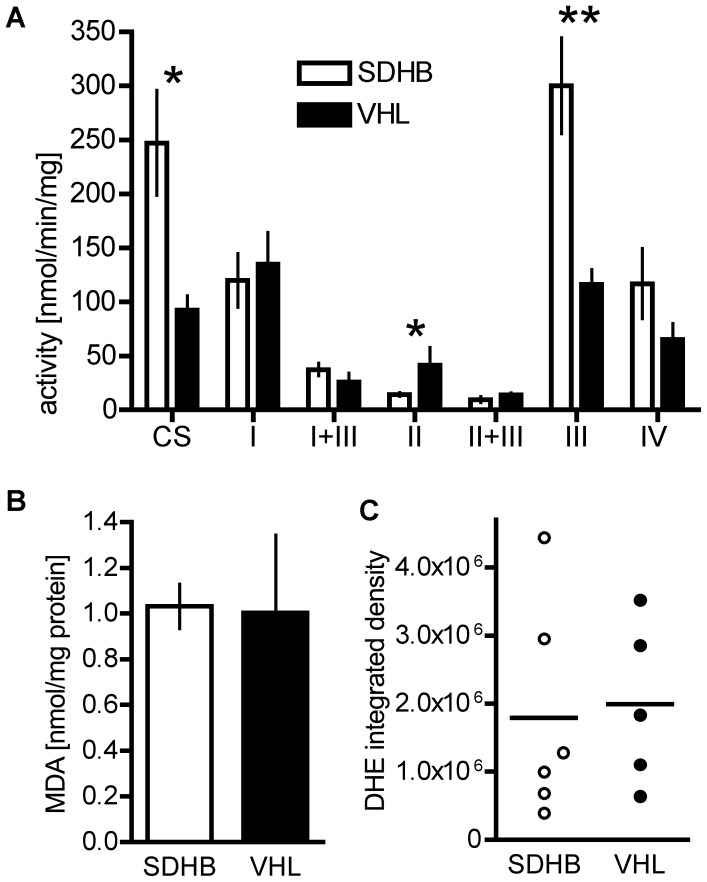
Tumor tissue levels of oxidative phosphorylation complex activity and oxidative stress. **A** Oxidative phosphorylation complex complex activity in SDHB- (n = 4) and VHL-derived (n = 4) PHEO/PGL tissue. * indicates p<0.05. **B** Malondialdehyde level in SDHB (n = 5) and VHL (n = 5) tissue as a measure of lipid oxidation. **C** Integrated density of DHE fluorescence in VHL (n = 5), SDHB (n = 6).

Activities of the OXPHOS complexes I-IV and CS were evaluated in VHL- (n = 4) and SDHB-derived primary PHEOs/PGLs (n = 2), as well as metastases (n = 2). No significant difference in the activities of any of the OXPHOS complexes was observed between SDHB-derived primary tumors and metastases; thus the samples were grouped together for comparison to VHL-derived PHEOs/PGLs. Comparison of VHL- and SDHB-derived samples revealed a higher activity of citrate synthase (CS) in SDHB in tissue homogenates (SDHB: 109.23±20.81 nmol/min/mg, VHL: 30.93±6.14 nmol/min/mg, p = 0.01) and isolated mitochondria (SDHB: 247.15±48.60 nmol/min/mg, VHL: 92.58±13.11 nmol/min/mg, p = 0.02) relative to total protein. In addition complex III activity was significantly increased in isolated mitochondria of SDHB-derived tumors (SDHB: 300.05±44.51, VHL: 116.28±13.56, p = 0.003) while complex II activity was decreased (SDHB: 14.30±1.97 nmol/min/mg, VHL: 41.40±16.65 nmol/min/mg, p = 0.045) (Fig. 5A).

### ROS Production in Human PHEOs/PGLs

Elevated SOD1 expression in MTT cells compared to MPC suggests a possible involvement of ROS in cell aggressiveness. Increased ROS production has been previously reported for cells with impaired SDHB [20], [32], [33], [34]. Thus, we evaluated levels of malondialdehyde as an index of lipid peroxidation, and dihydroethidium (DHE) fluorescence for SDHB- and VHL-derived tumor tissues (Fig. 5B, C). However, no differences could be observed between the different types of tissue.

Unlike in the cell model, in human tumor tissue no change in expression of the mainly cytosolic SOD1 (P00441) was observed when comparing VHL- and SDHB-derived PHEOs/PGLs. However, SOD2 (P04179) protein expression was increased in the SDHB-derived tumors (Fig. 4B).

## Discussion

Until recently the importance of Warburg’s observation of a shift to a glycolytic phenotype in tumor cells has often been underestimated. It is now becoming clear that aggressive tumors acquire dependence on glycolysis, often related to pseudo-hypoxia and/or OXPHOS dysfunction [35]. However, for PHEOs/PGLs pseudo-hypoxia is not an indicator of tumor aggressiveness: SDHB- and VHL-derived PHEOs/PGLs share a pseudo-hypoxic phenotype, while exhibiting quite distinct risks for the development of metastases.

In the present study, comparative analysis of MPC and MTT cells helped us to identify differentially expressed proteins characteristic of aggressive behavior. We chose this approach to avoid identifying differentially expressed proteins in direct comparisons of SDHB and VHL-PHEOs/PGLs that may be due to tumor location, the different mutations, or other factors that are not necessarily related to the aggressive behavior of SDHB tumors.

In both, MTT cells and SDHB-derived tumors, we found an elevated LDHA to LDHB ratio along with overall elevated LDH, which likely indicates imperfect coupling of glycolysis and the Krebs cycle in the comparably aggressive MTT cells and SDHB-derived tumors. In addition, we found evidence for aberrant OXPHOS: in MTT cells as evidenced by elevated ROS production at complex I and SOD1 over-expression; and in SDHB as evidenced by: an increased complex III activity, the expected decrease in complex II activity, and increased SOD2 expression.

LDH is a tetramer of A and B subunits, converting lactate into pyruvate and vice versa, depending on complex composition. Close coupling of glycolysis and OXPHOS is supported as long as the amount of LDHB outweighs or matches that of LDHA. However, with more of LDHA prevalent, decoupling with increased lactate production is promoted [36]. The ratio of LDHA to B was increased in MTT cells compared to MPC, while the ratio of LDHA to B was similar in human SDHB- and VHL-derived PHEOs/PGLs (Fig. 3). In VHL-derived PHEOs/PGLs there was a decrease in LDHB expression. In contrast, LDHA expression was increased in SDHB-derived PHEOs/PGLs. In conclusion, the conversion of pyruvate into lactate seems more likely than the reverse reaction in both SDHB and VHL-derived PHEOs/PGLs, possibly with a higher efficiency in SDHB-derived tumors due to a higher overall level of LDH. High cytosolic lactate levels have been reported to lead to an acidic tumor environment, presumably supporting aggressive tumors by protecting them from the host’s immune response and destabilizing the surrounding extracellular matrix, thereby enhancing tumor growth and spread (reviewed in [37]). Tumor-generated high lactate levels have also been shown to increase the risk of metastases or recurrence, and/or were correlated with reduced patient survival [38], [39], [40], [41]. Increased LDHA levels seem to be mandatory in tumor initiation as well as maintenance [42], [43]. Thus, targeting LDHA may be a promising therapeutic approach to treat cancers rich in LDHA, with presumed low systemic toxicity [44].

Favier and colleagues recently reported increased LDH activity in VHL- compared to non-VHL-PHEOs/PGLs. However, they did not report a significant difference in activity between VHL- and SDHx-PHEOs/PGLs [16]. Additional experiments on LDH enzyme activity or measurement of tumor lactate levels in fresh tumor tissue from SDHB- and VHL-PHEOs/PGLs will be of great interest for judging the functional impact of elevated LDHA levels in SDHB-PHEOs/PGLs.

Increased ROS production has been suggested to contribute to tumorigenesis in SDHB-derived tumors [20], [32], [33], [34]. Experimental evidence for increased ROS production due to dysfunction, inhibition, or down-regulation of other SDHx subunits is accumulating [45], [46], [47], [48], [49], [50]. Certain SDHx manipulations however did not cause ROS production [20], [51], [52]. Selak et al. elegantly showed that increased ROS are not required for HIF1α stabilization when SDHD is inhibited [53]. Nevertheless, SDHB- and SDHD-mutation induced increases in ROS have recently been shown to cause genomic instability that may contribute to carcinogenesis [33], [54]. While we did not directly observe a difference in cytosolic ROS levels and SOD1 expression between SDHB- and VHL-derived tumors, the mitochondrial SOD2 was more highly expressed in SDHB-related PGLs. Up-regulation of SOD2 has been suggested as a tumor cells’ escape from oxidative stress [55]. Aggressive tumor behavior has been previously associated with increased SOD2 expression, and seems to be mediated by the ensuing high H_2_O_2_ levels [56], [57]. Therapeutic targeting of SOD2 has been proposed to not only directly harm applicable tumor cells, but also to increase their susceptibility to ROS-inducing chemotherapeutic agents [58].

The DHE assay employed for ROS estimation in our study detects superoxide levels. Increased H_2_O_2_ levels, which may be expected when SOD2 is elevated, would thus be missed by this approach. In addition, analysis of lipid oxygenation and DHE reflect the oxygenation status of the whole cell, so it is not unlikely that mitochondrial ROS and/or H_2_O_2_ are elevated in SDHB- compared to VHL-PHEOs/PGLs.

To integrate our findings – which indicate decoupling of glycolysis and the Krebs cycle, with elevated lactate and H_2_O_2_ levels in the aggressive SDHB-PHEOs/PGLs – into the related energy metabolism pathways, we evaluated the expression of several glycolysis and OXPHOS genes in the human tumors, and in some cases in normal adrenal medulla. We confirmed an elevated GLUT1 and HK2 mRNA expression in VHL- relative to SDHB-PHEOs/PGLs [16] and put it into perspective to normal adrenal medulla. Elevated HK2 expression is considered a driving force towards a glycolytic phenotype [48]. Increased HK2 expression reportedly leads to enhanced proliferation and resistance to cell death in culture and correlates with poor prognosis in human glioblastoma multiforme [59]. Since VHL-PHEOs/PGLs have a better prognosis than SDHB-PHEOs/PGLs, the difference in HK2 does not seem to contribute to their distinct tumor aggressiveness in these particular tumors.

Previously, decreased OXPHOS activity and expression of selected OXPHOS complex subunits have been reported for VHL-derived PHEOs/PGLs [16], [60]. When comparing VHL- and SDHB-derived tumor tissues, in the latter we observed the expected decrease in complex II activity, but also an increase in complex III activity. In addition, we found the activity of CS, the first enzyme of the Krebs’ cycle, to be elevated in SDHB-derived tumors. Similar to the data presented by Goffrini et al. [33], our data indicates a selective decrease of complex II activity in SDHB-derived PGLs; however there is no evidence for complete disruption of OXPHOS.

Decreased OXPHOS activity in VHL-derived tumors has been proposed to be a result of decreased expression of several OXPHOS subunits, mediated by HIF and/or loss of p53/TIGAR [16]. In agreement with OXPHOS down-regulation in VHL, we observed significantly decreased mRNA expression of ND1 and Uqcrc1 compared to normal adrenal medulla. In addition, ND1, Uqcrc1, and CO1 were more highly expressed in SDHB-PHEOs/PGLs. Similarly, protein levels of NDUFB8, Uqcrc2, CO1, ATP synthase α and β, were increased in the majority of SDHB-derived PGLs. In contrast, Favier et al. [16] suggest decreased expression of selected OXPHOS proteins in SDHx-PHEOs/PGLs based on a limited number of SDHx-samples.

While we assume that the subunits we evaluated are crucial for the function of the respective complexes, we cannot exclude that they may not reflect the expression levels of other subunits of the same complexes and complex activity.

Our data confirm, to our knowledge for the first time in relation to normal adrenal medulla, the previously reported compliance of VHL-PHEOs/PGLs with the Warburg effect, i.e. increased expression of glycolysis- and decreased expression of OXPHOS-related genes [16], [21], [50]. In addition, our data suggests increased glycolysis and the decoupling of glycolysis and the Krebs cycle in SDHB-PHEOs/PGLs. However, our data show increased complex III activity as well as increased expression of all evaluated OXPHOS subunits in SDHB- compared to VHL-PHEOs/PGLs. In SDHB-PHEOs/PGLs, the mRNA levels of the evaluated OXPHOS subunits were at least as high as, or higher than those in normal medulla tissue. Thus, maintenance of OXPHOS in SDHB-PHEOs/PGLs, under circumvention of complex II, potentially connected to increased ROS production, seems plausible.

In the present study, findings from the differential expression analysis of the aggressive MTT cells compared to MPC guided our choice for the evaluation of key players in glycolysis and OXPHOS in human SDHB- and VHL-derived PHEOs/PGLs, as a first step in uncovering potential causes for increased tumor aggressiveness in SDHB-related tumors. Our data confirm that both SDHB- and VHL-related PHEOs/PGLs show features of a glycolytic phenotype, while presenting further support for the presence of distinct mechanisms of manifestation of the Warburg effect. In-depth analysis of the roles of elevated LDHA as well as elevated H_2_O_2_ production as a possible result of increased SOD2 expression may lead to a better understanding of these tumors and discovery of potential new therapeutic targets for the aggressive phenotype related to SDHB-mutations.

## Materials and Methods

### Ethics Statement

Tissue collection and genetic testing was performed with written patient consent under a protocol approved by the Eunice Kennedy Shriver National Institute of Child Health and Human Development Institutional Review Board.

### Human Samples

PHEO/PGL tissue was collected at the NIH and at Suburban Hospital in Bethesda, MD. A summary of patient information is given in Table 1. Genetic testing was performed following established guidelines for testing [61] under consideration of biochemical phenotypes. Human normal adrenal glands (n = 8), from 7 anonymous organ donors without any indication of adrenal dysfunction or tumor, were collected during radical nephrectomy or within 2–5 hours after confirmed brain death at the Department of Urology, School of Medicine, Comenius University, Bratislava, Slovakia. Adrenal medulla was separated from cortex and tissue identity was confirmed by qRT-PCR evaluation of chromogranin A and steroid acute regulator as described previously [62].

**Table 1:** Patient Information.

ID	Genetic backgr.	Age	Sex	Type	Location	Biochem.	OXP	DHE	MDA	WB	qRT-PCR
B01	SDHB	41	M	mm	spine	NA	x			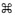	x
B02	SDHB	36	F	mm	A) Abdomen, R lower quadrant, lymph node B) lung	NA	x_A_			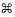 _A_	x_B_
B03	SDHB	35	F	mm	liver	NA		x			x
B04	SDHB	39	M	mm	R lung	NA		x			
B05	SDHB	16	M	pm	bladder	NA				*	
B06	SDHB	38	M	pm	abdomen (L suprarenal)	normal				*, 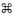	x
B07	SDHB	44	M	pm	retroperitoneal	NA				*, 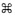	
B08	SDHB	33	M	pm	L retroperitoneal area	NA			x		
B09	SDHB^p^	23	M	pm	bladder	NA					x
B10	SDHB	10	F	mltp	L iliac bifurcation	NA & DA		x	x	*	
B11	SDHB	35	M	mltp	intra aortic	NA					x
B12	SDHB	31	M	sp	L peri-aortic mass, medial and caudal to kidney	DA	x	x	x	*	x
B13	SDHB	15	M	sp	retroperitoneal	normal				*, 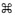	
B14	SDHB	30	F	sp	L adrenal	NA & DA		x	x	*, 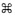	
B15	SDHB	13	M	sp	R periadrenal	nk				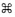	
B16	SDHB	53	M	sp	nasopharyngeal	NA & DA	x				x
B17	SDHB	47	F	sp	R adrenal	A & NA		x	x		
V01	VHL	32	M	bp	bilateral adrenal	NA				*, 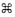	
V02	VHL	43	F	bp	R adrenal	NA				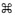	
V03	VHL	32	M	bp	L adrenal	NA		x	x	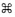	
V04	VHL	31	M	bp	bilateral adrenal	NA				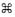	x
V05	VHL	39	F	bp	bilateral adrenal	NA & DA				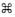	
V06	VHL	16	M	bp	bilateral adrenal	NA	x_L_	x_L_	x_L_	*_R_	x_L_
V07	VHL	28	M	bp	bilateral adrenal	NA		x_L_	x_L_	*_R_	
V08	VHL	13	M	bp	R adrenal	NA					x
V09	VHL	47	F	bp	bilateral adrenal	NA					x
V10	VHL	19	M	mltp	L adrenal	NA				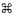	
V11	VHL	39	M	mltp	R adrenal	NA & DA				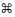	
V12	VHL	23	M	mltp	bilateral adrenal	NA & DA	x_R_	x_R_	x_R_	*_R_	x_L_
V13	VHL	23	M	mltp	carotid body	NA		x	x		
V14	VHL	52	F	mltp	R retrocaval mass	NA	x				x
V15	VHL	39	M	mltp	L para-aortic	NA	x			*	x
V16	VHL	39	F	sp	R adrenal	NA				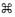	
V17	VHL^c^	48	F	sp	L adrenal	A				*	

ID (identifier): B followed by a number indicates SDHB, V followed by a number indicates VHL cases. Genetic Background: SDHB^p^: SDHB polymorphism, VHL^c^: VHL-Chuvash. Gender: F: female, M: male. Type: bp: bilateral primary, mm: metastatic metastases, mltp: multiple primary, pm: primary metastatic, sp: solitary primary. Location: R: right, L: left. Biochem. (biochemical phenotype): A: adrenergic, DA: dopaminergic, NA: noradrenergic, nk: not known. The 4 right columns indicate which samples have been used in the experiments specified by the column headings. Subscript letters are used if more than one tumor from the same patient was available, to indicate which sample has been used (R: right, L: left, A and B: as specified under the heading “location”. OXP (oxidative phosphorylation complex activity), DHE (dihydroethidium fluorescence), MDA (malondialdehyde), WB (western blot). In the western blot column, samples used for LDHA and B and LDHB blots are indicated by 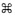. Samples used for all other proteins are indicated by *.

### Cell Culture

Mouse pheochromocytoma cells (MPC 4/30PRR) were a generous gift from Dr. Tischler, Tufts University, Boston. Their more aggressive filial MTT cells were generated and maintained as described by Martiniova et al. [31]. Briefly, cells of liver tumors, established in nude mice after tail vein injection of MPC, were cultured in bulk. The resulting MTT cells showed more aggressive behavior when re-injected than did their parental MPCs.

MPC and MTT cells were maintained at 5% CO_2_, 37°C in RPMI 1640 (Gibco, Grand Island, NY), supplemented with 10% heat-inactivated horse serum (Hyclone Logan, UT), 5% fetal bovine serum (Gibco), HEPES (Gibco), and penicillin (10,000 units/ml)/streptomycin (100,000 µg/ml) (Gibco). Medium was changed every other day and cells were passaged when 80–90% confluence was reached.

### 2D-sample Preparation

To harvest cells, they were incubated in 0.05% trypsin (Gibco) at 37°C until detachment. Then media was added and cells were collected by centrifugation at 3000×g for 3 min. Cells were washed in sterile 10 mM phosphate buffered saline (PBS; pH 7.4) and recollected by centrifugation three times.

Cell pellets were covered with 2D-lysis buffer (8 M urea, 4% CHAPS, 65 mM DTT, 40 mM Tris) and incubated on ice for 30 min. Suspensions were homogenized by sonication at 100 W for 30 seconds on ice. Samples were centrifuged at 20,800×g for 30 minutes. Protein from an aliquot of supernatant was precipitated with pre–cooled precipitation buffer (1∶1 ethanol: acetone containing 0.01% acetic acid) at a 1∶1 ratio, followed by incubation at −20°C over night. The protein pellet was washed in ethanol and dried in a SpeedVac before re-dissolution in 2D-lysis buffer. Protein concentrations were estimated using the Bradford assay (Bio–Rad, Hercules, CA, USA). Samples were kept at −80°C until analysis.

### 2D-gel Electrophoresis

Two-dimensional-gel electrophoresis and image analysis were performed as described by Jiang et al. [63]. Briefly, 80 µg protein of four replicate samples per group was separated in two dimensions by isoelectric focusing and sodium-dodecylsulfate-polyacrylamide-gel-electrophoresis (SDS-PAGE) for differential image analysis. Isoelectric focusing was performed using precast IPG dry strips, pH 3-10 NL (Amersham Biosciences, Uppsala, Sweden) in an IPG-Phor™ isoelectric focusing System (Amersham Pharmacia Biotech, Uppsala, Sweden) at a total of 60–70 kVh. After equilibration, IPG strips were placed on 12.5% polyacrylamide gels. SDS-PAGE was performed in a Hoeffer SE600 electrophoresis unit (Amersham Pharmacia Biotech). Protein was visualized by ammoniacal silver nitrate staining and gels were scanned using a GS–800 calibrated densitometer (Bio–Rad). PDQuest software (Bio–Rad) was used for differential expression analysis. Matched spots with differences in intensity * area in ppM>1.5, p<0.05 were chosen as spots of interest.

For protein identification, 2D protein separation was repeated with 1 mg total protein (0.25 mg of each replicate) under similar conditions. Protein was reversibly stained using Phastgel Blue R (Amersham Pharmacia Biotech). Proteins were reduced and alkylated with iodoacetamide prior to being loaded onto the gels.

### Protein Identification

Coommassie stained 2D-gel separated protein spots were excised and trypsin digested *in situ* using a modified version of the ABRF protocol (ABRF, *“In-Gel” Digestion Protocol for Proteins in SDS PAGE Gel.* 1997 (http://www.abrf.org/ABRF/ResearchCommittees/intprotseqrescomm.html)). Following digestion, peptides were extracted using the protocol above and reconstituted into 10 µl of 1∶1 acetonitrile: 0.1% TFA.

Initial mass spectrometric analysis was conducted using an ABI 4800 Proteomics Analyzer (Applied Biosystems, Framingham, MA) operated in reflector mode. Unseparated extracted peptide mixtures (0.5 µl) were applied with an equal volume of matrix, recrystallized HCCA matrix (0.5 mg/ml in 1∶1 acetonitrile: 0.1% TFA and 0.1 M NH_4_H_2_PO_4_) containing two internal mass standards, ACTH clip 18–39, MH^+^ = 2465.199, and the synthetic peptide RfffR (f = pentafluoro-phenylalanine), MH^+^ = 1042.276; in addition to the two internal standards added to the matrix, trypsin autolysis peptides were also used as internal calibrants. Reflector spectra consisting of 400 laser shots were obtained for each peptide mixture and inspected for candidate peptides for MS/MS analysis. Peptides selected for fragmentation analysis were chosen on the basis of both S/N intensity and separation from adjacent peptides using a Timed-Ion-Selector resolution of 300. MS/MS spectra consisting of 3000 laser shots were obtained under unimolecular decomposition conditions using a recently updated default calibration.

Proteins were detected using the Mascot [64] search algorithm for the fragmentation spectra of each sample using the following search constraints:

Fixed Cys modification: Iodoacetamide

Variable modification: Met oxidation

Peptide mass accuracy = 0.15 Da

Fragment mass accuracy = 0.06 Da

Charge state = +1

in the Swiss Prot database for Rodentia. Having detected a protein in this manner, its sequence was downloaded and used in conjunction with reflector peptide masses in the algorithm ProteinProcessor. This algorithm digests the protein sequence *in silico* with user-specified modifications to Met and Cys residues allowing one missed cleavage, then attempts to match observed peptide masses within a user-specified tolerance. The algorithm generates files of the digested protein and matched peaks. The latter includes a listing of the total reflector ion signal associated with standards and tryptic autolysis peaks along with the peptides matched against the candidate protein sequence, the accuracy, in ppm, of the match, modifications or missed cleavage and the percentage of sequence coverage along with fraction of total reflector signal; in addition, a listing of unmatched peaks is provided so that the likelihood of additional proteins being in the sample can be assessed. The program is available upon request as either a Perl script or a cgi file.

For some protein digests, the protein of origin could not be identified based on the MALDI spectra. For those, LC/MS/MS analysis was performed as described previously [65]. The resulting tandem mass spectra were extracted using BioWorks v3, and the MS/MS spectra were analyzed by both Mascot (version 2.3; Matrix Science, London, UK) [64] and X! Tandem (version 2007.01.01.2; www.thegpm.org) database search programs. For both search programs, the Sprot release 2010_10 (Nov 2, 2010) database (selected for Rodentia, 25,523 entries) was used with the fragment ion mass tolerance set to 0.60 Da and a 1.2 Da tolerance for the parent ion. The iodoacetamide derivative of cysteine was specified as a fixed modification, and oxidation of methionine was specified as a variable modification. Scaffold (version Scaffold 3_00_04, Proteome Software Inc., Portland, OR) was used to validate MS/MS based peptide and protein identifications. Peptide identifications were accepted if they could be established at greater than 95.0% probability as specified by the Peptide Prophet algorithm [66], and protein identifications were accepted if they could be established at greater than 99.0% probability and contained at least 3 identified peptides, using the Protein Prophet algorithm [67].

### Ingenuity Pathway Analysis

A list of the identified proteins together with their fold changes (Table S1) was analyzed through the use of IPA 6 (Ingenuity® Systems, www.ingenuity.com). Canonical pathways analysis identified the pathways from the IPA library of canonical pathways that were most significant to the data set. Proteins from the data set that met a fold change cutoff of 1.5 and were associated with a canonical pathway in the Ingenuity Knowledge Base were considered for the analysis.

### Western Blot

For western blot validation of proteins of interest, MPC and MTT cells were harvested as described above, using a different lysis buffer (10 mM 3-3-cholamidopropyl dimethylammonio 1-propane sulphate (CHAPS) in PBS, containing a Roche Minitablet protease inhibitor (1 tablet per 5 ml of PBS; Roche Applied Biosciences, Indianapolis, IN, USA)). After sonication, samples were centrifuged at 10,000×g for 10 min at 4°C. The supernatant was used as protein extract. Human PHEO/PGL tissue was homogenized on ice in the same lysis buffer. After homogenization the crude extracts were treated as described above for the cells. The protein concentration was estimated using the QuantIt protein assay (Molecular Probes Invitrogen, Carlsbad, CA, USA). Samples were diluted to the same concentration, containing 25% NuPage SDS loading buffer and 10% NuPage reducing agent. Proteins (20 µg) were separated in 4–12% Nu-PAGE Bis-Tris gels (Invitrogen) by reducing sodium-dodecylsulfate polyacrylamide gel electrophoresis (SDS-PAGE) and transferred to an Immobilon-P PVDF-membrane (Millipore, Billerica, MA, USA). To verify equal protein loading, membranes were stained using the MemCode Reversible Membrane Staining Kit (Pierce Biotechnology, Inc., Rockford, IL, USA). Membranes were blocked in 5% (w/v) nonfat dry milk, dissolved in 25 mM Tris buffered saline (TBS, pH 7.4) with 0.05% (v/v) Tween 20 (Sigma–Aldrich, St. Louis, MO, USA) (TBST) for 1 hour or over night. Antibodies were dissolved in 5% (w/v) Carnation nonfat dry milk (Nestlé USA, Solon, OH, USA) in TBST. Primary antibodies were left on membranes for 1 hour; secondary antibodies remained on membrane for 30 minutes. Before and after antibody incubations, membranes were washed in TBST three times for 5 minutes each. Primary antibodies were as follows: mouse-anti LDHB (Abnova, Walnut, CA, USA), rabbit-anti LDHA and B, rabbit-anti GALK1, mouse-anti ATP synthase β, rabbit-anti SOD1 (all Abcam, Cambridge, MA, USA), rabbit-anti ATP5C1 (ProteinTech Group Inc., Chicago, IL, USA), rabbit anti FLC1 (generous gift from Dr. Rouault, NICHD/NIH, Bethesda, MD, USA), mouse-anti Cytochrome C (Zymed, Invitrogen), goat anti actin (I-19) (Santa Cruz Biotechnology, Santa Cruz, CA, USA), MitoProfile® total OXPHOS rodent WB antibody cocktail (MitoSciences Inc., Eugene, OR, USA). Secondary antibodies were as follows: HRP conjugated donkey anti-mouse, donkey anti-rabbit and donkey anti-goat IgG (Santa Cruz). Antibody binding was visualized by incubation of the membrane in Super Signal West Pico Chemiluminescent reagent (Pierce Biotechnology) followed by exposure to Hyperfilm ECL (Amersham Biosciences GE Healthcare, Pistcataway, NJ, USA).

### Quantitative Real Time PCR

Samples of normal adrenal medulla (n = 4), SDHB- (n = 8), and VHL- (n = 7) derived PHEOs/PGLs were used. Tissue RNA extraction, reverse transcription and qRT-PCR were performed as previously described [62]. Quantitation of genes of interest as well as *18S* as internal control was performed using the 7500 RT PCR System and Taqman Gene expression assays (Hs02596864_g1 CO1, Hs00892681_m1 GLUT1, Hs00175976_m1 HK1, Hs00606086_m1 HK2, Hs00929956_m1 LDHB, Hs00855332_g1 LDHA, Hs02596873_s1 ND1, Hs00987255_m1 PKM2, Hs00163415_m1 UQCRC1, Applied Biosystems, Foster City, CA, USA). Relative expression of target genes to *18S* was calculated based on the delta delta Ct method. Weighted one way ANOVA, with Student-Newman-Keuls correction for post hoc comparisons was used to test statistical significance overall and then various between group comparisons using Stata (Release 12, StataCorp, College Station, Texas, USA).

### Mitochondrial Oxygen Consumption and ROS Production (Permeabilized Cells)

Cells were suspended in medium containing 125 mM sucrose, 65 mM KCl, 2.5 mM KH_2_PO_4_, 1 mM MgCl_2_, 20 µM EGTA, 20 mM HEPES, 0.1% Bovine serum albumin, pH 7.4, and 5 mM glutamate/malate or succinate (in the presence of 2 µM rotenone) as substrates and permeabilized with digitonin (30 µg/ml). Oxygen consumption was determined polarographically using a Clark oxygen electrode (World Precision Instruments, Sarasota, FL, USA) in the basal state (S4) and after the addition of 600 µM ADP (S3).

ROS production was measured fluorometrically in permeabilized cells in an assay based on the detection of hydrogen peroxide generated during substrate catabolism in a horseradish peroxidase (HRP) coupled reaction using 10-acetyl-3,7-dihyrdoxyphenoxazine (Amplex Red reagent, Molecular Probes, Invitrogen). 10^6^ cells were incubated in the respiration medium and the ROS deriving from the mitochondrial respiratory chain were determined after the addition of either 5 mM glutamate/malate or succinate (in the presence of 2 µM rotenone) as substrates. The velocity of H_2_O_2_ production is calculated from a calibration curve obtained adding known amounts of H_2_O_2_. All assays were performed at 37°C on instruments equipped with thermostatic control and magnetic stirring. Significance of differences was calculated using Welch’s version of the 2-tailed unpaired t-test in Prism 4 (GraphPad Software Inc., La Jolla, CA, USA).

### Tumor OXPHOS Complex Activity

Frozen tumor tissue was homogenized and mitochondria were isolated according to standard differential centrifugation procedures [68] in 150 mM KCl, 10 mM Tris/HCl, 2 mM EDTA and 2 µg/ml aprotinin (pH 7.4) at 4°C. All samples were stored at −80°C.

Activities of respiratory chain complexes NADH:coenzyme Q10 reductase (complex I), succinate:coenzyme Q10 reductase (complex II), succinate:cytochrome c reductase (complex II+III), NADH: cytochrome c reductase (complex I+III), coenzyme Q10:cytochrome c reductase (complex III), and cytochrome c oxidase (complex IV) as well as citrate synthase (CS) were measured spectrophotometrically at 37°C in tissue homogenate or isolated tissue mitochondria as described in [69]. Differences between the groups were evaluated by the t-test on the logarithms of the measured activities, using Stata.

### Oxidative Stress (Tissue)

Oxidative stress in the tumor tissue was determined by malondialdehyde (MDA) formation measurement and DHE staining. MDA, an end product of lipid peroxidation, was determined spectrophotometrically by measurement of 2-thiobarbituric acid reactive substances (TBARS), as described previously [70]. For DHE staining, 3 consecutive 10 µm thick cryosections of each tumor sample were incubated with 5 µmol/l DHE (Sigma) for 30 minutes at 37°C, washed twice with PBS, mounted and visualized using a confocal microscope (Zeiss, LSM 510 Meta). Significance of differences was calculated using Welch’s version of the 2-tailed unpaired t-test in Prism 4.

## Supporting Information

Table S1 **Protein identification summary.** Table listing the identified proteins for each of the spots of interest and identification characteristics. In addition, fold changes of spot intensities between MPC and MTT cells from the preparative gels are listed. Fold changes listed here together with the protein IDs were used for Ingenuity Pathway Analysis.
(XLSX)

Table S2 **Peptide identification summary.** Table listing the characteristics of detected peptides for each spot, including their sequences and database search specifics.
(XLS)
